# Topographic Cues Impact on Embryonic Stem Cell Zscan4-Metastate

**DOI:** 10.3389/fbioe.2020.00178

**Published:** 2020-03-06

**Authors:** Carlo F. Natale, Tiziana Angrisano, Luigi Pistelli, Geppino Falco, Viola Calabrò, Paolo A. Netti, Maurizio Ventre

**Affiliations:** ^1^Interdisciplinary Research Centre on Biomaterials, University of Naples Federico II, Naples, Italy; ^2^Department of Biology, University of Naples Federico II, Naples, Italy; ^3^Department of Chemical, Materials and Industrial Production Engineering, University of Naples Federico II, Naples, Italy; ^4^Center for Advanced Biomaterials for HealthCare@CRIB, Istituto Italiano di Tecnologia, Naples, Italy

**Keywords:** embryonic stem cell, Zscan4, topography, cell adhesion, cytoskeleton

## Abstract

The extracellular microenvironment proved to exert a potent regulatory effect over different aspects of Embryonic Stem Cells (ESCs) behavior. In particular, the employment of engineered culture surfaces aimed at modulating ESC self-organization resulted effective in directing ESCs toward specific fate decision. ESCs fluctuate among different levels of functional potency and in this context the *Zscan4* gene marks the so-called “metastate,” a cellular state in which ESCs retain both self-renewal and pluripotency capabilities. Here we investigated the impact of topographic cues on ESCs pluripotency, differentiation and organization capabilities. To this aim, we engineered culturing platforms of nanograted surfaces with different features size and we investigated their impact on ESCs multicellular organization and *Zscan4* gene expression. We showed that the morphology of ESC-derived aggregates and *Zscan4* expression are strictly intertwined. Our data suggest that ESC Zscan4 metastate can be promoted if the adhesive surface conditions guide cellular self-aggregation into 3D dome-like structure, in which both cell-material interactions and cell-cell contact are supportive for *Zscan4* expression.

## Introduction

The interactions between cells and the extracellular environment, either native or artificial, strongly affect cell behavior (Gattazzo et al., 2014; Ventre and Netti, 2016; Smith et al., 2018). In this regard, embryonic stem cells (ESCs) that are isolated from blastocysts, make no exception (Guilak et al., 2009). ESCs are a major focus in regenerative medicine since they promise to provide an essentially unlimited supply of cells for transplantation. ESCs are usually maintained in culture on feeder layers in the presence of biochemical supplements to sustain self-renewal (Keller, 2005). A number of different protocols have been developed to guide the differentiation of ESCs and the large majority rely on the generation of embryoid bodies (EBs) comprising the three embryonic germ layers (Itskovitz-Eldor et al., 2000). ESCs fluctuate among different levels of functional potency which are spontaneously induced, in regular *in vitro* culture conditions, as a consequence of cell-to-cell interactions and paracrine effects (Hisada et al., 2012; Jiang et al., 2013). Such ESCs heterogeneity in culture is reflected by a gene signature that marks the so-called metastate, a specific sub-population state in which ESCs retain both self-renewal and pluripotency capabilities (Zalzman et al., 2010). Recent findings show that *Zscan4*, Zinc finger, and SCAN domain protein 4, is a key pluripotency factor that marks ESC subpopulation that is referred to as a high-level of pluripotency metastate. Although only a small fraction of undifferentiated ESCs expresses *Zscan4* at any given time, undifferentiated ES cells oscillate, i.e., change expression levels in a transient and reversible manner, between a *Zscan4* negative (Zscan4−) and positive (Zscan4+) state. Remarkably, Zscan4+ metastate can be enhanced by retinoic acid (RA) and is characterized by the lack of canonical pluripotency genes *Oct3/4* and *Nanog* while retaining self-renewal and pluripotency capabilities (Sharova et al., 2016; Tagliaferri et al., 2016). Global expression profiling revealed that Zscan4-metastate is characterized by the expression of key markers of ectodermic lineage thus suggesting that it is primed to early ectoderm. Therefore, *Zscan4* marked metastate represents an ideal *in vitro* model to monitor cell fate decisions dynamically and a valuable opportunity to analyze the transition between pluripotency and early ectodermal specification. Owing to their extraordinary characteristics of pluripotency and self-renewal, controlling ESCs functions through external stimuli is of crucial importance for potential therapeutic applications, as this would bypass gene- or drug-induced manipulations. Beside their potential therapeutic applications, ESCs are also employed *in vitro* for the generation of tissues or organoids, which constitute valuable models for studying tissue/organs development, the dynamics of diseases or as testing platforms for drug screening and discovery (Fatehullah et al., 2016; Lancaster and Huch, 2019). In order for the ESCs to initiate and sustain the organogenesis process, a plethora of exogenous stimuli need to be adequately provided to the system (Yin et al., 2016). So far, our ability to initiate and sustain ESC self-organization and differentiation is mostly based on the use of ill-defined substrate properties and on the presentation of morphogens in a scarcely controlled manner, which together limit the reproducibility and efficacy of the entire process (Huch et al., 2017; Kratochvil et al., 2019). Yet, a recent literature proved that specifically engineered materials displaying well-defined biochemical and biophysical stimuli such as surface topography, material stiffness and wettability directly influence ESC lineage specification and self-organization (Chowdhury et al., 2010; Mei et al., 2010; Chen et al., 2012; Eroshenko et al., 2013). Integrating biochemical supplements with material-derived stimuli would add another level of control on ESC fate that could eventually improve the efficiency of differentiation, the self-organization into histologically competent systems and ultimately enhance the reproducibility of the process (Kratochvil et al., 2019). Altogether these aspects may pave the way toward the development of novel engineering concepts for functionalizing surfaces aimed at gaining better control on stem cell functions and fate. This might find useful applications in a variety of contexts including, but not limited to, functional scaffold for tissue engineering or the development of devices that control the differentiation state of ESC populations.

We have recently demonstrated that nanotopographic patterns are able to guide the self-organization and differentiation of Mesenchymal Stem Cells (MSCs) into tendon-like structures whose histological and molecular profiles are reminiscent of embryo tendons (Iannone et al., 2015). Here, we employed a transgenic murine ESC line (ES^Zscan_Em^ cells) where Zscan4 expression is marked by the Emerald green fluorescence protein reporter, enabling us to characterize Zscan4+, in living cell cultures (Zalzman et al., 2010). To understand the impact of topographic cues on ESCs pluripotency, differentiation, and organization capabilities, we cultured ES^Zscan_Em^ cells on polydimethylsiloxane (PDMS) nanograted or flat elastomeric substrates to investigate the impact of topographic patterns on *Zscan4* gene expression by image analysis and gene expression analyses.

## Materials and Methods

### Preparation of Patterned Substrates

Patterned substrates were obtained by means of replica molding. In particular, commercially available optical media supports were processed as described by Anene-Nzelu et al. and then used as masters for fabricating the elastomeric patterned substrates (Anene-Nzelu et al., 2013). Two types of masters were used, one with ridge: grove width of ~350 nm (termed ps-350), the other with ridge: grove width of ~700 nm (termed ps-700). PDMS (Sylgard 184, Dow Corning) was prepared by mixing the elastomer base and curing agent at a 10:1 weight ratio. The solution was degassed, poured onto the masters and then cured at 60°C for 2 h. Control (flat) PDMS substrates were produced by pouring the base and curing mix on a 35 mm polystyrene petri dish (Corning) and curing at 60°C for 2 h. Then patterned and control PDMS substrates were cut into circular pieces of 9.6 cm^2^. The structural features of the replica surfaces were characterized by means of scanning electron microscopy (SEM) and atomic force microscopy (AFM). For SEM examinations, the substrates were mounted on microscope stubs and sputter-coated with gold (Sputter Coater 208 HR, Cressington, Watford, UK). Images were acquired with a Zeiss Ultra Plus FESEM scanning electron microscope (Oberkochen, Germany) using an accelerating voltage of 10 kV. For AFM imaging, the surfaces of nanogrooved samples were scanned with a NanoWizard atomic force microscope (JPK Instruments, Berlin, Germany). Images were acquired in contact mode using a silicon nitride tip with a nominal spring constant of ~0.1 N m^−1^ (MSCT, Bruker, Billerica, MA, USA). The scanning area was set at 30 × 30 μm, and images were recorded at a line-scan rate of 1 Hz in the air at room temperature. The preparation for cell culture first included UV sterilization for 1 h. Then, to increase PDMS hydrophilicity, substrates were treated with ozone UV deep light (Berdichevsky et al., 2004). The treatment was performed with a ProCleaner Plus for 1 h. Finally, to improve ESC adhesion on the PDMS, surfaces were incubated with a gelatin 0.1% solution for 15 min prior to cell culturing experiments (Fu et al., 2017).

### Cell Culture

Murine ES^Zscan_Em^ transgenic cells were derived from the inner cell mass of mouse (strain 129P2/OlaHsd) blastocyst. The cell line of origin is the E14Tg2a.4 ES (ATCC, Manassas, VA, USA) (Tagliaferri et al., 2016). Cells were cultured for two passages on gelatin-coated feeder-free plates and subsequently maintained in gelatin-coated six-well plates in complete ES medium: GMEM (Glasgow Minimal Essential Medium, Sigma-Aldrich, St. Louis, MO), 15% FBS (EuroClone, Milan, Italy), 1,000 U ml-1 leukemia inhibitory factor (LIF) (EuroClone, Milan, Italy), 1.0 mM sodium pyruvate (Invitrogen, Carlsbad, CA, USA), 0.1 mM non-essential amino acids (Invitrogen, Carlsbad, CA, USA), 2.0 mM L-glutamine (Invitrogen, Carlsbad, CA, USA), 0.1 mM β-mercaptoethanol (Sigma-Aldrich, St. Louis, MO), 500 U ml-1 penicillin/streptomycin (Invitrogen, Carlsbad, CA, USA) and 5.0 mM blasticidine (Sigma-Aldrich, St. Louis, MO). ESCs were incubated at 37°C in 5% CO_2_. Cell culture medium was changed daily and cells were split every 2–3 days routinely. All-trans retinoic acid (RA) was dissolved in DMSO. 2·10^4^ ES^*Zscan*_*Em*^ were plated on patterned and control substrates in the medium supplemented with 1.5 μM RA. For differentiation, negative controls of patterned substrates are represented by ES^Zscan_Em^ transgenic cells cultivated on plastic dishes either in presence of regular medium containing 0.1% DMSO (C-RM) or supplemented with RA (C-RA). Morphological, immunofluorescence and molecular analyses were performed at 72 h post-seeding.

### Time-Lapse Experiments

The temporal evolution of ESC colony formation and growth was assessed by means of time-lapse microscopy. Briefly, experiments started 1 h after cell seeding on patterned and control substrates. Cell populated samples were placed in an incubator (Okolab, Naples, Italy) mounted on the stage of an Olympus IX81 microscope. At least six regions per substrate were randomly acquired in bright field and in epifluorescent mode for 72 h at 2 frame/h rate. Images were collected with a 10× objective lens (numerical aperture 0.3) and were acquired with a digital ORCA- Flash 2.8 camera (Hamamatsu). In these experiments, ESCs were observed for 72 h after RA addition.

### Immunofluorescence

Cells cultured on patterned and control substrates were fixed 72 h after seeding with 4% paraformaldehyde (Sigma) in PBS for 15 min. Cells were then permeabilized with 0.1% Triton X-100 (Sigma Aldrich-Merck KGaA, Darmstadt, Germany) in 1× PBS (TPBS). Samples were blocked in 3% bovine serum albumin in PBS (Sigma-Aldrich) for 1 h to avoid non-specific binding. E-Cadherin was labeled by incubating samples with an anti-E-Cadherin monoclonal antibody (dilution 1:250, Abcam) for 1 h at 20°C. After incubation, substrates were washed three times with TPBS (3 min per wash) and incubated with Atto 646-conjugated goat anti-mouse antibody (dilution 1:600, Sigma) for 1 h at 20°C. Actin filaments were stained by incubating samples with Alexa Fluor™ 594 phalloidin (dilution 1:250, ThermoFisher Scientific) for 1 h at 20°C. Samples were thoroughly rinsed in PBS and mounted on glass slides by using mounting media (Pro-Gold anti-fade). Fluorescent images of Zscan4-Emerald-GFP, E-Cadherin, and actin bundles were collected with a Zeiss confocal microscope LSM-710 (Carl Zeiss). Samples were excited at 488 nm (Zscan4), 545 (actin) and 640 (E-Cadherin) laser lines, and the emissions were collected in the 500–530, 550–600, and 660–690 nm ranges, respectively. Moreover, Z-stacks were acquired with the optimal interval suggested by the software, followed by the application of a maximum intensity algorithm.

### Quantitative Real Time-PCR

2·10^4^ ES^Zscan_Em^ cells/cm^2^ were grown for 72 h on ps-350, ps-700 and Flat patterned substrates in regular medium (RM) supplemented with 0.015% DMSO or 1.5 μM RA (Sigma-Aldrich, St. Louis, MO). Quantitative Real Time-PCR (qPCR) analysis was performed according to di Martino et al. (2016). Total RNA was isolated from cells of each well using TriReagent (Invitrogen, Carlsbad, CA, USA) according to the manufacturer's instructions. RNA (1 μg) was used to generate reverse-transcribed cDNA using the Retro cDNA Synthesis Kit (Bioline Meridian Bioscience Inc., London, UK). qPCR with SYBR green (SsoAdvanced™ Universal SYBR® Green Supermix, Bio-Rad, Hercules, CA, USA) detection was performed with a 7500 RT-PCR Thermo Cycler (Applied Biosystem, Foster City, CA, USA). Expression levels were normalized using Gapdh mRNA expression. The relative levels of mRNA were calculated using the 2^−ΔΔCT^ method. Experiments were all performed in triplicate. The sequences of gene-specific qPCR primers are reported in Table 1.

**Table 1:** Primers used for qPCR analysis.

**Gene**	**Primer forward**	**Primer reverse**
*Zscan4*	5′-AGTCTGACTGATGAGTGCTTGAAGCC-3′	5′ -GGCCTTGTTGCAGATTGCTGTTG-3′
*Prame*	5′-TGCTGCCAAATTCCTTTCTC-3′	5′-GAGAGTTGGCAGCGATTCAT-3′
*tcstv1*	5′-ATCCTCAGGAACTGAGAACTTCTGG-3′	5′-ATCCCATTCGGCAATCCAGC-3′
*Nanog*	5′-AACCAGTGGTTGAAGACTAGCAATGGTC-3′	5′-TTCCAGATGCGTTCACCAGATAGC-3′
*Oct4*	5′-CCGTGTGAGGTGGAGTCTGGAGAC-3′	5′-CGCCGGTTACAGAACCATACTCG-3′
*Rex1*	5′-CAGAAGAAAGCAGGATCGCCTCAC-3′	5′-GCCACTTGTCTTTGCCGTTTTC-3′
*Foxa2*	5′-CTGGGAGCCGTGAAGATGGAAG-3′	5′-TCCAGCGCCCACATAGGATG-3′
*Gata2*	5′-AGCTCATGACTATGGCAGCA-3′	5′-CCGGTTCTGTCCATTCATCT-3′
*Neurod1*	5′-GCTCCAGGGTTATGAGATCG-3′	5′-CTCTGCATTCATGGCTTCAA-3′
*Gapdh*	5′-AATGGTGAAGGTCGGTGTG-3	5′-GAAGATGGTGATGGGCTTCC-3′

### Image Analysis

Colonies area and Zscan4-Eme fluorescent intensity were evaluated with the command Analyze Particles of Fiji. At least 20 confocal digital images were randomly collected and analyzed for each substrate from two independent experiments. The confocal pinhole was set in order for the optical slice to include the entire signal emitted by ESC colonies. Images were processed using the threshold command (method “triangle”). The normalized Zscan4 fluorescent intensity was calculated using the following formula:

$$\begin{array}{l} \frac{\sum I_{Zscan4+}}{A_{Zscan4+}} \end{array}$$

where ∑*I*_*Zscan*4+_ is the sum of the signal intensity of the pixels of the cells expressing Zscan4 (Zscan4+) cells and *A*_*Zscan*4+_ is the area occupied by Zscan4+ cells.

The fraction of Zscan4+ cells is defined as the ratio of *A*_*Zscan*4+_ with the total area occupied by cells. In symbol,

$$\begin{array}{l} \frac{A_{Zscan4+}}{A_{ESC}} \end{array}$$

where *A*_*ESC*_ is the area of the ESC population.

The normalized E-cadherin fluorescence intensity quantification was calculated with Fiji using the following formula:

$$\begin{array}{l} \frac{\sum I_{E-Cadh}}{A_{E-Cadh}} \end{array}$$

where ∑*I*_*E*−*Cadh*_ is the sum of the signal intensity of the pixels of the cells expressing E-cadherin and *A*_*E*−*Cadh*_ is the area occupied by a cell expressing E-cadherin.

The profile of the fluorescence intensities of Zscan4-Eme and E-Cadherin along the thickness of the cell aggregates were reported as follows: on the x-axis, 0 value referred to apical plane whereas 1 corresponded to basal one. On the y-axis, the fluorescent intensity values of each region of interest were normalized with the maximum intensity attained in that region of interest in order to obtain intensity values in the 0–1 interval.

### Statistical Analysis

Significant differences of data obtained from image analysis were assessed by ANOVA test followed by Tukey's multiple comparisons post-test written in Matlab.

Statistical analyses of the qPCR data were performed using GraphPad Prism6. Statistical significance of the difference in measured variables between control and treated groups was determined ANOVA test followed by Tukey's multiple comparisons post-test. Raw data of the image analyses and Real Time-PCR are reported in Data Sheet 1.

## Results

### Topographic Guidance Affects ESC Colonies Morphology and Zscan4 Activation

**Figure 1:**
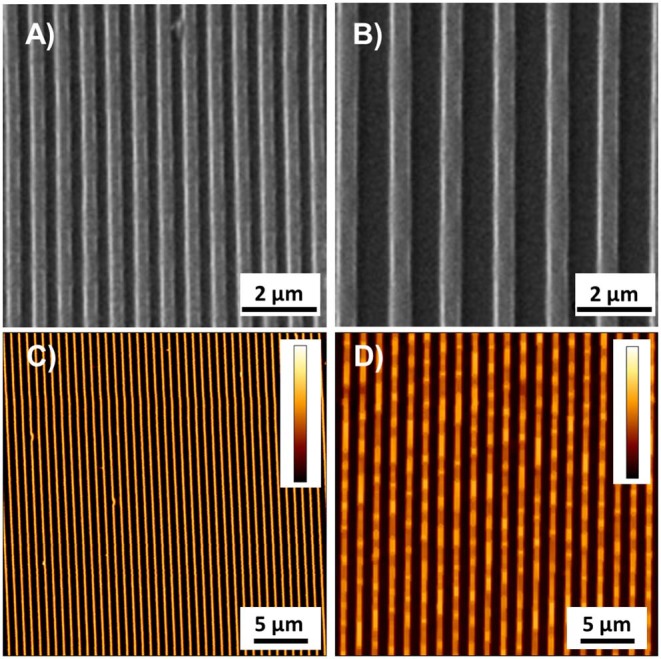
Representative images acquired with SEM and AFM of ps-350 **(A,C)** and ps-700 **(B,D)** patterned surface. Colorbar represents the interval 0–150 nm in **(C)** and 0–255 nm in **(D)**.

**Figure 2:**
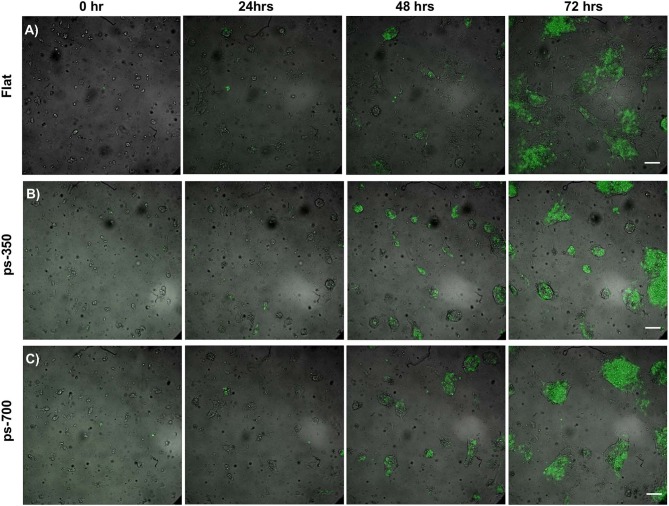
Topographic signals impact ESC metastate. Time-lapse images of ESCs cultured for 72 h on flat control **(A)** and nanograted **(B,C)** surfaces expressing Zscan4-Emerald green. Fluorescent signal (green) is superimposed onto the transmission image (grayscale). Scale bar is 100 μm.

The experimental set-up was designed in order to manipulate the biophysical environment of ESC cultures *in vitro* and to assess its role in ESC fate decision. In particular, topographic signals with different feature dimensions were presented to cells and we assessed their effect on the *Zscan4* gene expression, along with other relevant markers of pluripotency and differentiation. We first characterized the morphology of the nanostructured surfaces. Representative images acquired with SEM and AFM of the PDMS patterned surfaces are reported in Figure 1. The images show a groove and ridge widths of ~350 nm and groove depth of ~150 nm on ps-350 (Figures 1A,C), whereas and groove and ridge widths of ~700 nm and groove depth of ~250 nm on ps-700 (Figures 1B,D). We have previously demonstrated that substrates displaying topographic features within this range effectively confine focal adhesion formation and growth ultimately affecting cytoskeleton arrangement and cell alignment both at single cell and multicellular level (Natale et al., 2014; Iannone et al., 2015; Coppola et al., 2018). In order to assess the effect of topographic signals on ESC Zscan4 metastate, we monitored the expression of *Zscan4* gene in cells grown for 72 h on control or nanopatterned surfaces by means of time lapse microscopy (Figure 2). Initially, ES^*Zscan*_*Em*^ transgenic cells were isolated or grouped into small clusters on all type of substrates and bright green fluorescent spots, i.e. Zscan4+ cells, were occasionally detected. A clear green fluorescent signal was evident from 12 h onwards. At 48 h post-seeding, on control surfaces, ES^*Zscan*_*Em*^ cells grew as flattened colonies divided by isolated cells (Figure 2A, Video 1). Clusters of cells were observed on nanopatterned surfaces. Such clusters increased in size forming dome-like structures and the vast majority of them contained fluorescent cells (Figures 2B,C, Videos 2, 3). At 72 h post-seeding, flat substrates were almost entirely covered by shallow cellular aggregates, whereas ES^*Zscan*_*Em*^ cells formed large and isolated dome-shaped colonies on ps-350 substrates. An intermediate behavior was observed on ps-700 substrates.

**Figure 3:**
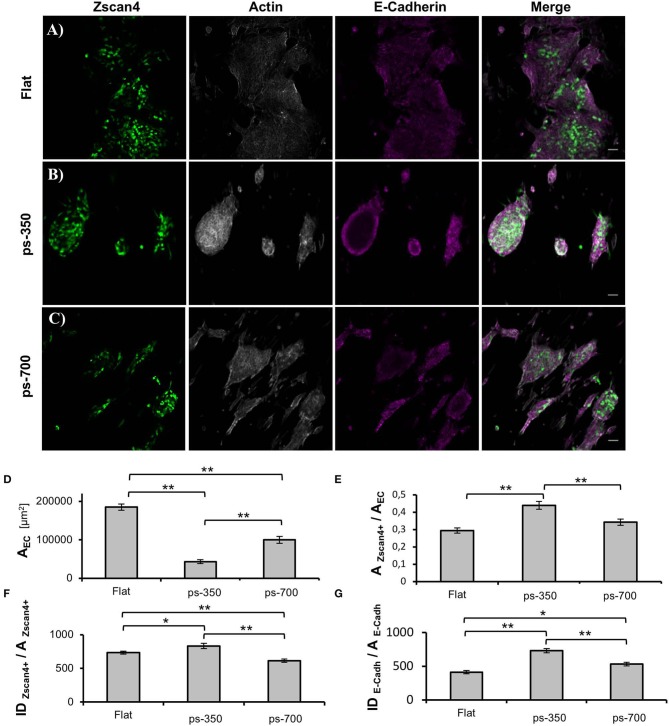
Nanotopographic cues impact ESC colony morphology. Zscan4, actin cytoskeleton and E-cadherin confocal images of ESCs cultured on flat control **(A)**, ps-350 **(B)**, and ps-700 **(C)** surface for 72 h. Scale bar is 50 μm. Histograms of projected area of the ESC colonies **(D)**, fraction of the area occupied by Zscan4+ cells within ESC colonies **(E)**, ES^*Zscan*_*Em*^ fluorescence intensity values normalized with respect to the area occupied by Zscan4+ cells **(F)** and E-cadherin fluorescence intensity values normalized with respect to the area occupied by cells expressing E-cadherin **(G)** of ESC cultured for 72 h on flat (*n* = 20), ps-350 (*n* = 20), and ps-700 (*n* = 20) surfaces. Data are mean ± SEM. Asterisk (*) indicates statistically significant differences between groups (*p < 0.05; **p < 0.01).

To gain a better insight into the three-dimensional organization of the supracellular structures, we fixed the cultures at 72 h and stained them for actin and E-cadherin. Figures 3A–C display *Zscan4* expressing cells randomly distributed within the aggregates, irrespective of the underlying substrate. However, on flat substrates, ES^*Zscan*_*Em*^ cells defined extended patches covering a large proportion of the surface area, while ES^*Zscan*_*Em*^ cells grown on patterned substrates were contained in well-defined structures. These results were consistent with our bright-field microscopy observations. Moreover, the aggregates on ps-700 substrates were predominantly oblate in shape with their major axis parallel to the direction of the pattern. However, on the flat and ps-350 substrate, they did not show any preferential direction of growth or alignment. The E-Cadherin signal was always observed within the supracellular aggregates. While the structures grown on flat substrates displayed a rather uniform E-Cadherin signal, on patterned substrates the dome-like structures displayed E-cadherin signal predominantly at the peripheral regions (Figure S1).

We next performed image analysis to assess whether the aggregates grown on patterned substrates contained more Zscan4+ cells or expressed higher levels of *Zscan4*. First, we calculated the projected area of the supracellular aggregates from images displaying the actin signal. The aggregates grown on ps-350 were significantly smaller than those on flat surfaces (Figure 3D) while on ps-700 the size of aggregates was intermediate. Then, we measured the fraction of the aggregate area occupied by Zscan4+ cells. Remarkably, Zscan4+ cells covered a significantly higher fraction of the aggregates area on patterns compared to that measured on flat substrates. In particular, aggregates grown on ps-350 reached the highest values of Zscan4+ area (Figure 3E).

We then asked whether the different surface patterns could influence the *Zscan4* gene expression level, in other words, if the fluorescence intensity emitted by Zscan4+ cells was dependent on the type of substrate. Since larger projected areas are expected to contain more cells, we normalized the fluorescence intensity value with the area occupied by Zscan4+ cells. We found that structures formed on ps-350 not only contained the highest fraction of Zscan4+ cells but they also emitted significantly higher fluorescence with respect to structures formed on ps-700 or flat substrates (Figure 3F) thereby indicating that the ps-350 pattern stimulates and potentiates *Zscan4* gene expression. Using a similar procedure, we also measured the intensity of E-cadherin signal. As shown in Figure 3G, we found that structures formed on ps-350 emitted significantly higher E-cadherin fluorescence compared to structures formed on ps-700 or flat substrates.

### Topographic Cues Impact Actin Cytoskeleton Organization, Zscan4 and E-Cadherin Spatial Positioning

**Figure 4:**
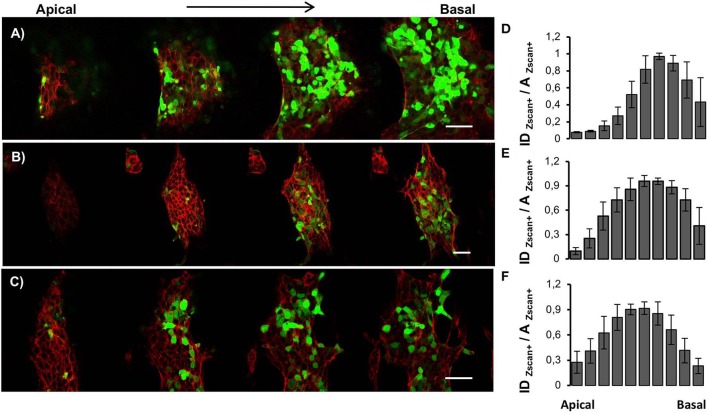
Spatial organization of Zscan+ cells within ESC colonies. Confocal z-scan imaging of actin cytoskeleton (red) and Zscan4 (green) of ESCs cultured for 72 h on flat control **(A)**, ps-350 **(B)**, and ps-700 **(C)** surfaces. Fluorescence intensity distribution profiles along with the thickness of ESCs cultured for 72 h on flat **(D)**, ps-350 **(E)**, and ps-700 **(F)** surfaces. For each experimental condition, 8 confocal z-scanning acquisitions were analyzed. Data are mean ± SD. Scale Bar is 50 μm.

**Figure 5:**
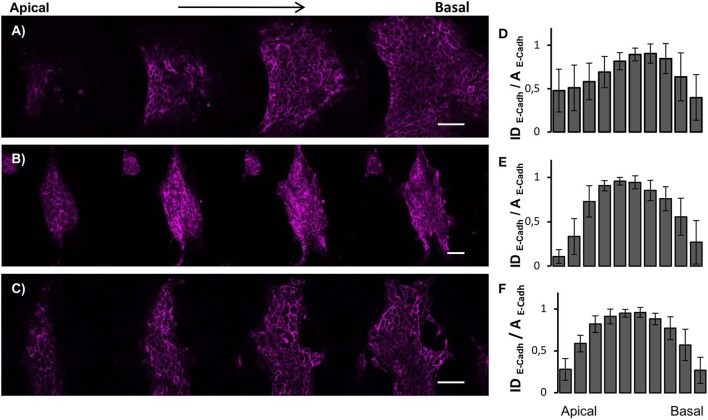
Spatial organization of cell-cell contacts within ESC colonies. Confocal z-scan imaging of E-Cadherin and corresponding fluorescence intensity distribution profiles of ESCs cultured for 72 h on flat control **(A,D)**, ps-350 **(B,E)**, and ps-700 **(C,F)** surfaces. For each experimental condition, 10 confocal z-scanning were analyzed. Data are mean ± SD. Scale Bar is 50 μm.

Since ESCs aggregates are three-dimensional organoid-like structures, we performed Z-scan confocal imaging to better analyze the spatial organization of Zscan+ cells (Figures 4A–C). By scanning the structures from the apical plane down to the basal plane, we observed local progressive changes in the actin organization. Structures grown on flat surfaces possessed a well-spread and shallower morphology (Figure 4A). Moreover, well-structured actin fibers retracing ESCs contours were detected on flat control surfaces both in the apical and the basal plane (Figure S2A). Conversely, structures grown on patterned surfaces possessed prevalently a globular shape and a transition in actin fibers organization between apical and basal plane was detected (Figures 4B,C). In fact, in the apical and mid strata of the structures, actin was mostly cortical, retracing cell contour of each of them. However, approaching the basal plane, stress fibers crossing the cell body became clearly visible (Figures S2B,C). Next, we calculated and plotted the fluorescence intensity of Zscan4+ cells (normalized with respect to the area occupied by Zscan4+ cells) according to the relative axial (z-axis) position (Figures 4D–F). On the flat surfaces, the intensity profile was skewed toward the basal plane (Figure 4D). In this case, high Zscan4 levels were expressed in cells located close to the surface. However, on the patterned substrates, the profile of fluorescence intensity was bell-shaped, almost symmetrical, suggesting that the more intense fluorescence came from the mid-planes (Figures 3E,F). Thus, cells expressing high Zscan4 levels are mostly located in the central portion of the organoid-like structures. To verify whether the spatial pattern of expression of E-cadherins correlated with that of Zscan4, we performed an E-cadherin signal quantification in the axial direction. Image analysis revealed that the spatial pattern of expression of the E-cadherin followed a trend similar to that of Zscan4 (Figure 5). More specifically, the higher E-cadherin signal intensity in structures grown on flat substrates was found in cells located close to the substrate (Figures 5A,D). Conversely, higher fluorescence intensity was observed by the mid cross-section of the dome-like structures grown on patterned substrates (Figures 5B,C,E,F).

### Topographic Patterned Surfaces Affect ESC Differentiation Dynamics

Given the different morphology of ESC structures obtained on the three different growth surfaces and their differential level of Zscan4 promoter activation, we tested if this phenomenon was a sign of a different cell fate commitment. To address this point, we cultured ES^*Zscan*4−*Eme*^ transgenic cells on flat, ps-350 and ps-700 surfaces in the presence of RA. C-RM and C-RA were used as negative controls of patterned substrates. After 72 h of treatment, ESCs acquired the different growth patterns above described.

**Figure 6:**
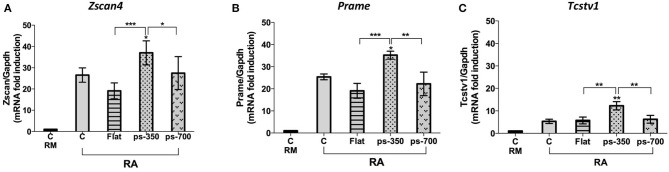
Zscan4 metastate enrichment requires canonical RA induction and specific micropattern plate of growth. The ES^Zscan_Em^ cells were cultured for 72 h on plates control CTR (RM and RA), Flat, ps-350, and ps-700 surfaces treated with RA. mRNA expression levels of Zscan4-Metastate genes: Zscan4 **(A)**, Prame **(B)**, Tcstv1 **(C)** were assessed by qPCR and normalized to RM condition. Error bars represent SD of triplicate samples from three independent biological replicates. Asterisks over the ps-350 column indicate significant difference with respect to the CTR-RA condition. Asterisks over the square brackets indicate significant differences between groups. Levels of significance are: ns > 0.05; *p < 0.05; **p < 0.01; ***p < 0.001.

To examine the association of aggregate morphology with the metastate marked by *Zscan4* positive expression, we analyzed the expression levels of genes such as *Prame* and *Tcstv1* that are well-known markers of this metastate along with endogenous *Zscan4* itself. qPCR experiments were performed to measure *Zscan4, Prame, Tcstv1* mRNA levels on the different micropattern plates, after RA induction. Results shown in Figure 6A indicate that *Zscan4* expression was significantly higher on ps-350 compared to ps-700 and Flat surfaces thus confirming the results obtained by immunofluorescence (Figure 3F). Accordingly, also *Prame* and *Tcstv1* were up-regulated on ps-350 (Figures 6B,C).

**Figure 7:**
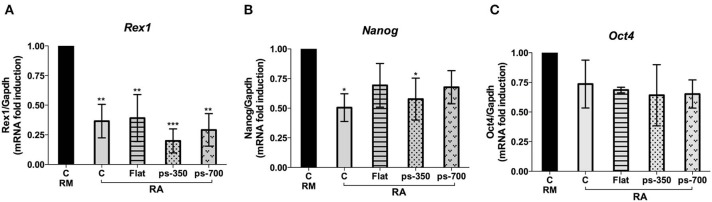
RA induces ES-Zscan4 cell differentiation. The ES^Zscan_Em^ cells were cultured for 72 h on control surfaces (CTR, RM and RA), Flat, ps-350, and ps-700 surfaces treated with RA. mRNA expression levels of pluripotency genes: Rex1 **(A)**, Nanog **(B)**, and Oct4 **(C)** were assessed by qPCR and normalized to RM condition. Error bars represent SD of triplicate samples from three independent biological replicates. Asterisks indicate significant differences with respect to the CTR-RM condition. Levels of significance are: ns > 0.05; *p <0.05.

Concerning the panel of canonical pluripotency genes we investigated, i.e., *Rex1, Nanog* and *Oct4* we found a general reduction of their expression according to the induction of Zscan4 metastate by RA (Tagliaferri et al., 2016). The expression of *Rex1* was particularly sensitive to the biophysical microenvironment, as we observed a significant downregulation with respect to the control for all the substrates (Figure 7A). A marked downregulation was recorded in the case of cells cultivated on the ps-350 surfaces Also *Nanog* expression was significantly downregulated on ps-350, whereas *Oct4* showed a general, although non-significant, downregulation on all the tested substrates (Figures 7B,C).

**Figure 8:**
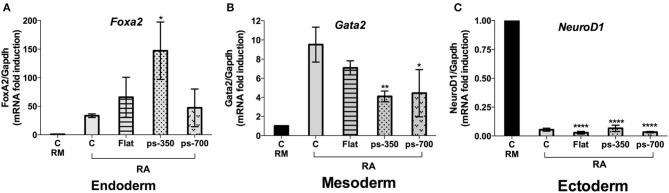
The Zscan4 expression is required for endodermal differentiation. The ES^Zscan_Em^ cells were cultured for 72 h on control plates (CTR, RM and RA), Flat, ps-350, and ps-700 treated with RA. mRNA expression levels of differentiation genes: Foxa2 (endoderm) **(A)**, Gata2 (mesoderm) **(B)**, and Neurod1 (ectoderm) **(C)** were assessed by qPCR and normalized to RM condition. Error bars represent SD of triplicate samples from three independent biological replicates. Asterisks indicate significant differences with respect to the CTR-RA condition. Levels of significance are: ns > 0.05; *p < 0.05; **p < 0.01; ****p < 0.0001.

To evaluate a possible specific effect of patterned substrates on differentiation commitment, we measured *Foxa2, Gata2* and *Neurod1* gene expression as biomarkers of an endodermal, mesodermal and ectodermal lineage, respectively. We found a 3.0-fold enrichment of *Foxa2* gene expression on structures grown on ps-350 compared to those grown on ps-700 substrates (Figure 8A) with a corresponding reduction of *Gata2* gene expression (Figure 8B). The increase of *Foxa2* gene expression along with the enrichment of Zscan4-metastate in cells grown on ps-350 indicates that RA and the 350 nm micro-pattern cooperate to drive ESCs toward the endodermal differentiation fate. Finally, *Neurod1* gene expression was repressed in presence of RA regardless of the patterned substrate used thereby indicating that patterned surfaces do not influence the inhibition of ectodermal lineage commitment induced by enrichment of Zscan4+ cell population (Figure 8C).

## Discussion

The extracellular microenvironment proved to exert a potent regulatory effect on ESCs fate decision. In particular, the delicate balance existing between cell-matrix interactions and cell-cell aggregation plays a pivotal role during embryogenesis. For example, cell junctions are required during the compaction of the inner cell mass whereas interactions with extracellular matrix components occur later during the inner cell mass formation at blastocysts preimplantation stage (Leivo et al., 1980;Kemler et al., 1989).

*In vitro*, features of the culturing materials that regulate cell adhesion and spreading, including substrate stiffness, topography, and ligand density, proved to be effective in directing fate decisions. The molecular mechanisms governing the transduction of material signals into the expression of specific genes is not thoroughly clear. However, a growing number of works suggests that substrates depressing adhesion, like soft or nanorough substrates or those exhibiting a low density of ligands, favor self-renewal (Chowdhury et al., 2010; Jeon et al., 2012; Murray et al., 2013), whereas material features improving adhesion and contractility, like stiff substrates or with a high density of ligands, promote ESC differentiation (Evans et al., 2009; Murray et al., 2013). Along these lines, substrates displaying topographic patterns introduce an additional level of control on cell adhesion as topographic relieves may define the localization and orientation of adhesion plaques, which finely affect cell spreading and elongation. Kingham et al. showed that the square pattern of nanopits directed hESCs mesodermal differentiation in the absence of soluble, chemical induction factors (Kingham et al., 2013). Using nanoscale patterns of parallel grooves, Lee et al. induced the formation of hESC colonies of elongated cells that spontaneously differentiated in neurons (Lee et al., 2010). These data suggest that specific arrays of topographic signals can possibly overcome the requirement for soluble factors to govern the cell differentiation program.

In this work, we provide new evidence on the effects of surface topography on the self-organization and lineage specification of ESCs through a gene reporter model. Our results show for the first time the correlation between the nanopatterned support and the pluripotency ground state marked by the expression of *Zscan4*. In particular, the system we employed is suitable to monitor the genomic reprogramming of ESCs through a live approach by a direct measure of a fluorescent signal. Zscan4 marked metastate represents a cellular state in which ESCs retain wide pluripotency capabilities (Zalzman et al., 2010). More in detail, *Zscan4* is specifically expressed at 2-cell stage embryos and in a small fraction (1–5%) of ESCs population (Falco et al., 2007; Storm et al., 2009) characterized by a high level of pluripotency since it produces both embryonic and extra-embryonic cell derivatives (Macfarlan et al., 2012). Here, we found that the simple presence of a topographic signal is not sufficient to promote cell self-organization and direct fate decision, but rather a specific feature of the patters that culminate with the formation of dome-shaped organoid-like systems.

Our findings show that morphology of ESC-derived aggregate and *Zscan4* expression are strictly intertwined. The formation of supracellular aggregates is the result of a delicate balance between cell-cell and cell-material interactions, i.e., cells must be sufficiently anchored to the substrates but also sufficiently mobile to enable clustering (Powers et al., 1997). To modulate cell-substrate adhesion, we used PDMS substrates. This has been extensively used as substrate for stem cell culture owing to its chemical stability, ease of functionalization and compatibility with micro- and nano-fabrication technologies (Kuddannaya et al., 2013; Chuah et al., 2015). For instance, Eroshenko et al. showed that fibronectin-coated PDMS not only supports ESCs attachment and proliferation but also its stiffness affects fate decisions as mesodermal differentiation was upregulated on stiff substrates (Eroshenko et al., 2013). Similarly, Evans et al. demonstrated that increasing PDMS substrate stiffness upregulates gene expression of mesendodermal markers and terminal osteogenic differentiation of ESCs (Evans et al., 2009). Rather than stiffness, here we affected adhesivity through nanotopographic patterns. Although both ps-350 and ps-700 display the same accessible area for cells to adhere (total area of ridges), focal adhesions that form on the ps-350 substrates are expected to be smaller, which impairs cell adhesion. Therefore, it is likely that cell generated forces are sufficiently high to locally detach cells from the substrate thus producing dome-like structures. We reported a similar behavior and dynamics of self-organization of human MSCs on a PDMS substrate with an analogous topography (Natale et al., 2014; Iannone et al., 2015). Our results show that the formation of the dome like-structures is accompanied by extensive cell-cell interactions (Figure 3F) and a reduction, but not annihilation, of cell-material adhesion (Figure 3D). Together these aspects correlate with an enhanced expression of *Zscan4* suggesting that ps-350 substrates promote the formation of a microenvironment that favors *Zscan4* expression. Cells on flat substrates can form adhesions everywhere and these can grow in any direction. This is likely to promote firm adhesion of cells to the substrates, which was confirmed with larger and shallower ESC colonies. In terms of adhesivity, the ps-700 substrate provides an intermediate level of adhesion, which results in morphologies and gene expression patterns that are in between those observed for ps-350 and flat substrates.

Remarkably, our data are consistent with studies reporting that topographic features in the size range we adopted disrupt integrin-binding reducing colony spreading (Biggs et al., 2010). Moreover, Ghanian et al. demonstrated that Poly(ε-caprolactone) nanofibrous substrates affect hESC colonies morphology, with 200 nm fiber diameters resulting in the formation of 3D spheroid structures providing a better environment for definitive endoderm differentiation (Ghanian et al., 2015). Furthermore, intercellular interaction within ESC colonies was shown to regulate pluripotent and self-renewal signaling pathways with E-cadherin-mediated intercellular adhesion ultimately affecting ESCs survival and cloning (Larue et al., 1994; Chen et al., 2010).

Here we show that the decreased ESC attachment/spreading on the 350 nm substrate was associated with cell-cell aggregation and a consequent enhancement of E-cadherin expression along the peripheral regions of the dome-like structure. Such a phenomenon was much less evident on flat surfaces, whereas on ps-700 substrates we observed an intermediate behavior. Similar behavior was observed by Ji et al. where spherical ESC-derived colonies formed on 400 nm silica colloidal crystal (SCC) possessed the strongest expression for E-cadherin at the periphery of the colonies (Ji et al., 2012).

Within such a peculiar 3D organization our findings strongly indicate that dome-like structures might constitute a favorable environment for the *Zscan4* expression. Indeed, dome-like structures cultured on 350 nm surfaces not only contain the highest fraction of Zscan4+ cells but they also emitted significantly higher fluorescence signals with respect to structures formed in other conditions. This phenomenon was observed by both immunofluorescence image analysis and genetic assessment and in both cases, *Zscan4* was up-regulated when ESCs were organized into the organoid 3D-like structure. Consistently, along with *Zscan4* up-regulation other metastate markers, *Prame* and *Tcstv1*, resulted up-regulated when ESCs were cultivated on ps-350 surfaces. Since Tagliaferri et al. demonstrated that retinoic acid supplementation induces Zscan4 metastate in about 20% of the ESC population (Tagliaferri et al., 2016), our data indicate that such percentage can significantly be increased by appropriate adhesive surfaces such as those triggering cellular self-aggregation into 3D organoid-like structure where well-packed ESCs are connected one to another.

Finally, in line with previous reports, we found that the activation of *Zscan4* correlates with the expression of endodermal differentiation markers. In fact, our data showed that the synergistic effect of RA and nanopatterned surfaces drive ESCs toward the endodermal differentiation.

In conclusion, we provided further evidence that material stimuli in the form of nanotopographic patterns affect the supracellular self-organization of ESCs into complex and spatially organized structures. Material signals can in principle be exploited to regulate the balance between cell-substrate adhesion and cell-cell interactions thus affecting ESC fate decisions. Patterned substrates can, therefore, be employed to direct and guide spheroids or possibly organoids *in vitro*, without resorting to complex functionalization schemes.

## Data Availability Statement

All datasets generated for this study are included in the article/Supplementary Material.

## Author Contributions

CN, TA, VC, and MV designed the study. CN, TA, and LP performed the experimental work and analyses. CN, TA, VC, GF, PN, and MV contributed technical support and discussion. CN, TA, VC, and MV wrote and edited the manuscript.

### Conflict of Interest

The authors declare that the research was conducted in the absence of any commercial or financial relationships that could be construed as a potential conflict of interest.

## Supplementary Material

Supplementary Data Sheet 1 Spreadsheet of image analyses and Real Time-PCR raw data.
