# Supplementary material for: Topographic Cues Impact on Embryonic Stem Cell Zscan4-Metastate
Source: Front Bioeng Biotechnol. 2020 Mar 6;8:178. doi: 10.3389/fbioe.2020.00178 (PMC7069379; doi:10.3389/fbioe.2020.00178)
Supplement: Supplementary file 1 [file Table_1.docx]

**SUPPORTING INFORMATION**

**Topographical cues impact on Embryonic Stem Cell Zscan4-metastate**

Carlo F. Natale ^1,^, Tiziana Angrisano^2^, Luigi Pistelli^2^, Geppino Falco^2^, Viola Calabrò ^2^, Paolo A. Netti^1,3,4^ and Maurizio Ventre * ^1,3,4^

*^1^Interdisciplinary Research Center in Biomedical Materials (CRIB), University of Naples Federico II, Naples 80125, Italy*

*^2^Department of Biology, University of Naples Federico II*

*^3^Department of Chemical, Materials and Industrial Production Engineering and Interdisciplinary Research Centre on Biomaterials, University of Naples Federico II, Italy*

*^4^Center for Advanced Biomaterials for HealthCare@CRIB, Istituto Italiano di Tecnologia, Naples, Italy*


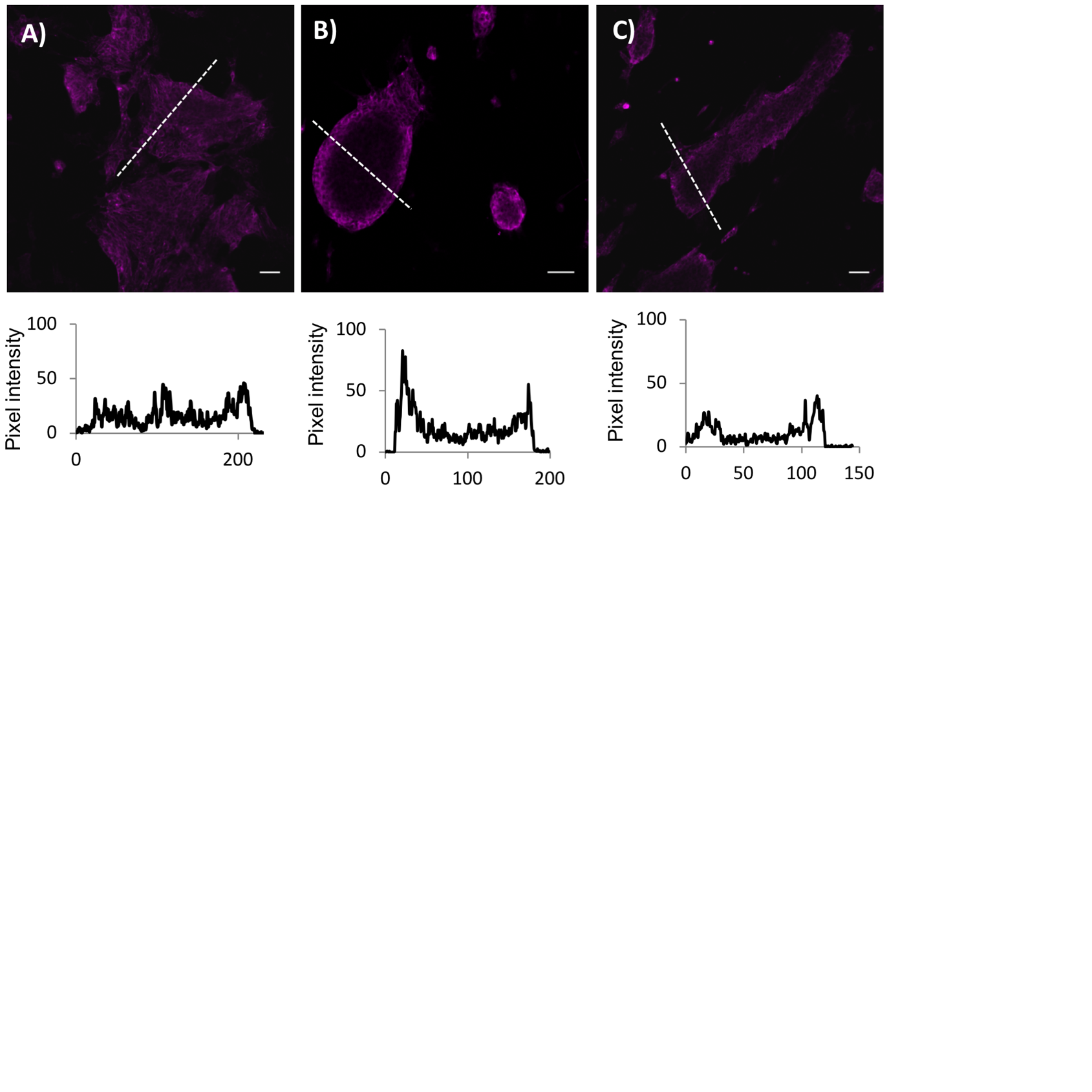


Figure S1: E-cadherin confocal images corresponding fluorescence intensity distribution profiles of ESCs cultured on flat control (a), ps-350 (b) and ps-700 (c) surfaces for 72 hours. Scale bar is 50 µm.


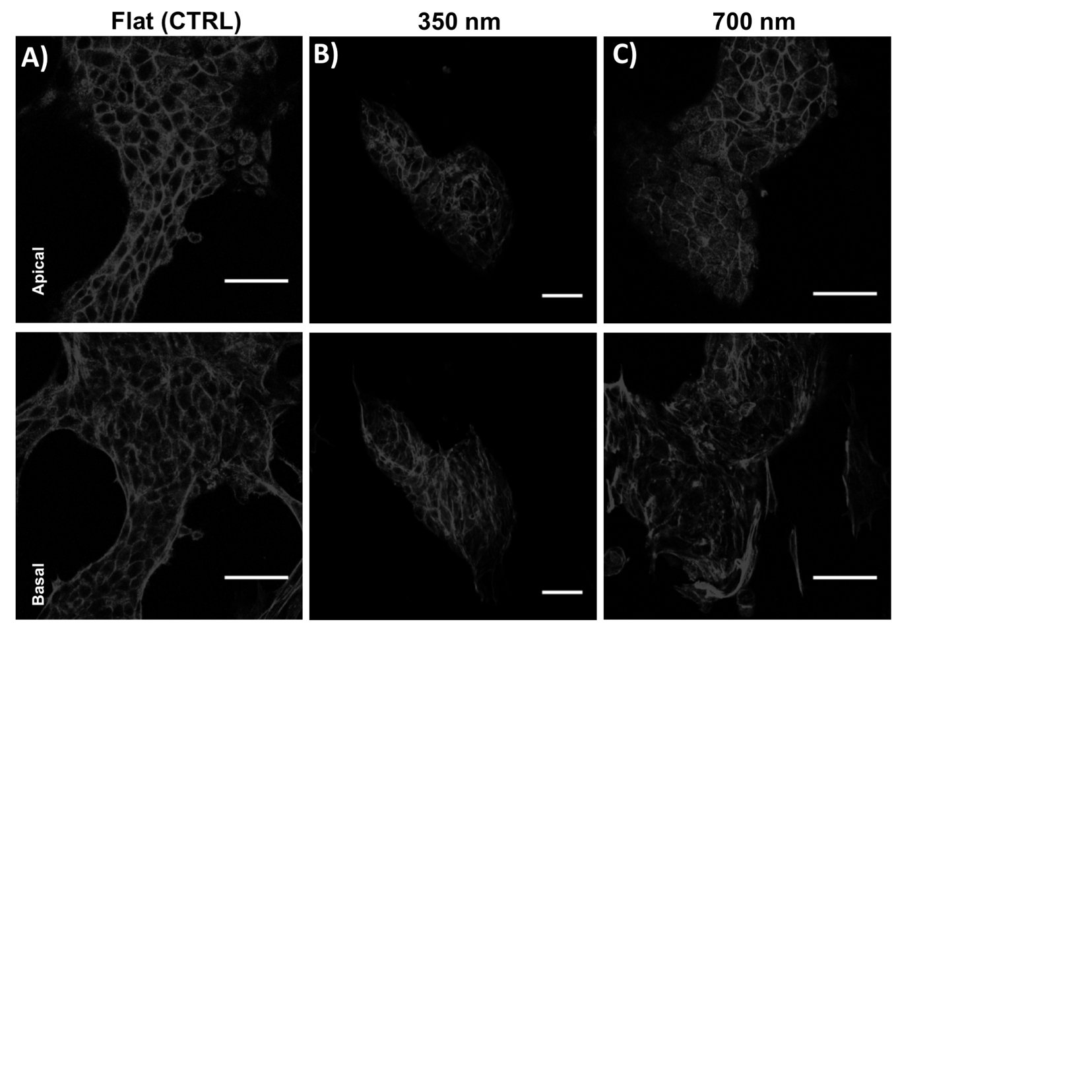


Figure S2: Confocal z-scan imaging of apical and basal actin cytoskeleton of ESCs cultured on flat control (a), ps-350 (b) and ps-700 (c) surfaces for 72 hours. Scale bar is 50 µm.

**VIDEO CAPTURES**

**SuppVideo-S1**: Time lapse videos of ESCs cultured for 72 hours on control surface expressing Zscan4-Emerald green fluorescence protein reporter superimposed onto the transmission image (grayscale). Scale bar is 100 µm

**SuppVideo-S2**: Time lapse videos of ESCs cultured for 72 hours on ps-350 surface expressing Zscan4-Emerald green fluorescence protein reporter superimposed onto the transmission image (grayscale). Scale bar is 100 µm

**SuppVideo-S3**: Time lapse videos of ESCs cultured for 72 hours on ps-700 surface expressing Zscan4-Emerald green fluorescence protein reporter superimposed onto the transmission image (grayscale). Scale bar is 100 µm
